# The real experience of nurse role reconstruction under the implementation of the no-attendant care policy in Shanghai hospitals: a qualitative study

**DOI:** 10.3389/frhs.2025.1663682

**Published:** 2025-10-03

**Authors:** Yuhan Cheng, Sibei Wan, Yifan Jiang, Yi Sheng, Qian Wu, Yan Shi, Li Wang

**Affiliations:** ^1^ School of Medicine, Tongji University, Shanghai, China; ^2^ Department of Nursing, Shanghai Tenth People's Hospital Affiliated to Tongji University, Shanghai, China; ^3^ Department of Nursing, Shanghai General Hospital Affiliated to Shanghai Jiao Tong University, Shanghai, China

**Keywords:** companion-free hospitalization, nurse role reconfiguration, phenomenological qualitative research, patient safety, health workforce policy

## Abstract

**Objective:**

To explore nurse role reconstruction under the implementation of no-attendant care services in Shanghai hospitals, and to identify challenges and strategies for optimization.

**Methods:**

A phenomenological qualitative design was adopted. From June 1 to July 1, 2025, semi-structured face-to-face interviews were conducted with 14 registered nurses from no-attendant care wards in three tertiary hospitals in Shanghai. Data were transcribed verbatim and analyzed using Colaizzi's seven-step method. Two researchers independently coded, discrepancies were resolved through discussion and methodological arbitration, and results were validated through member checking to ensure rigor.

**Results:**

Four main themes and eleven subthemes were extracted: (1) Expanded Responsibilities and Intensified Workload: Significant Increase in Work Intensity; New Challenges in Professional Competence; Multidimensional Accumulation of Occupational Stress. (2) Positive Impacts on Patients and Families: Effective Relief of Family Care Burden; Enhanced Perceived Value of High-Quality Nursing. (3) Challenges in the Implementation Process: Blind Spots in Patient Safety Management; Need to Improve Nurse–Patient Communication Efficiency; Inadequate Mechanisms for Accountability. (4) Optimization Strategies: Establish a Legal Safeguard System; Improve Insurance Payment Coordination Mechanism; Strengthen Training Mechanisms for Nursing Assistants. A few minority or dissenting perspectives were incorporated as contextual modifiers.

**Conclusion:**

No-attendant care improves patient experience and alleviates family caregiving burden, but simultaneously places heavier physical, risk, and relational demands on nurses, highlighting the complexity of role reconstruction. This study demonstrates the essential features of nurses’ lived experiences under this model and emphasizes the need for systemic support, such as legal frameworks, insurance coverage, and workforce training, to optimize care delivery and sustain professional resilience.

## 1 Introduction

In China, family caregivers of hospitalized patients commonly face considerable financial, temporal, and emotional pressures. Many must take leave from work or travel long distances, rest inadequately at the bedside, and assume complex care tasks without systematic training. This not only increases the risk of improper practices and caregiver fatigue, but may also create patient safety hazards and disrupt family functioning (1). For families seeking cross-regional care, expenses for transportation and lodging further amplify these burdens. In tertiary hospitals in Shanghai, characterized by complex case mixes and rapidly changing conditions, both patients and families increasingly demand nursing that is “professional, continuous, and accessible” (2). How to reduce reliance on informal family care while ensuring safety and quality has become an urgent issue for clinical management and nursing practice.

Against this backdrop, some hospitals have begun exploring “full-spectrum daily care” delivered by nurses and certified care workers during hospitalization. Under this model, medical institutions take primary responsibility for defining service content, ensuring staff qualifications, and controlling quality, while family members shift from continuous bedside presence to informed decision-making and remote communication (3). This approach relieves families of time and financial pressures, while concentrating more basic care, health education, communication, and risk monitoring within the nursing team, thereby reshaping role boundaries, workloads, and competency structures (4). At the clinical frontline, this has created new tensions: care tasks have become more intensive, communication chains lengthier, and accountability more complex, while also promoting a return to fundamental nursing and renewed recognition of nursing's value.

At the policy level, directional support has been provided. First, the Action Plan for Further Improving Nursing Services (2023–2025) called for optimizing service delivery and promoting model innovation based on patient needs, offering both policy space and quality guidance for inpatient comprehensive care (5). Second, the National Pilot Program for No-Attendant Inpatient Care (2025) set explicit goals and baseline requirements, emphasizing informed consent, standardized services, and information transparency to promote standardization and homogeneity (6). Third, the Basic Standards for No-Attendant Inpatient Care Services (T/SHNA 0012-2025) issued by the Shanghai Nursing Association exemplifies local operational guidance, detailing service content, staff training, and fee disclosure, and incorporating services into quality control systems to support implementation (7). These policies have provided a framework for developing no-attendant care, while simultaneously pushing the practical transformations faced by frontline nurses into focus.

However, existing studies have primarily concentrated on model construction and outcome evaluation, with little attention paid to the lived experiences of nurses in the no-attendant care context, particularly the specific challenges, stress mechanisms, and coping strategies associated with role reconstruction (8). To address this gap, the present study adopted a phenomenological qualitative approach, targeting nurses from no-attendant care wards in tertiary hospitals in Shanghai. The study aimed to explore changes in work content, professional experiences, and adaptive strategies during implementation, with the goal of providing frontline evidence to inform nursing workforce allocation, competency development, legal and insurance coordination, and overall service improvement.

## 2 Methods

### 2.1 Study design

This study adopted a phenomenological qualitative design. Semi-structured face-to-face interviews were conducted to gain an in-depth understanding of how nurses, as one of the primary implementers of no-attendant care services, experienced role changes, encountered challenges, and developed coping strategies during the implementation process. (In this study, “full-spectrum daily care” refers to hospital-organized provision of daily living and basic nursing care during hospitalization, including personal hygiene, feeding and oral care, toileting and elimination assistance, repositioning and early mobilization, vital sign monitoring, basic rehabilitation and health education, as well as remote communication with families).

### 2.2 Participants and recruitment

A purposive sampling strategy was employed to recruit nurses from no-attendant care wards in three tertiary hospitals in Shanghai (the highest level in China's hospital classification system). Inclusion criteria were: (1) registered nurses employed in the hospital; (2) working in a ward where no-attendant care services were implemented; and (3) providing informed consent and willingness to participate. Exclusion criteria were: (1) nurses who had not yet provided care for patients under the no-attendant model; and (2) student nurses or probationary nurses.

Potential participants were contacted via email or WeChat and invited to join the study. Following the principle of maximum variation, participants were enrolled sequentially until thematic saturation was achieved. In total, 14 nurses were recruited, all of whom provided informed consent and participated fully in the interviews. Basic demographic characteristics of participants are presented in Table 1.

**Table 1:** Demographic and professional characteristics of the participating nurses.

ID	Age	Job title	Education	Competency level	Years of experience	Department
HuN1	38	Senior Nurse	Bachelor's	N4	16	Neurology
HuN2	27	Nurse	Associate	N2	7	Respiratory Medicine
HuN3	43	Senior Nurse	Bachelor's	N3	22	Cardiology
HuN4	46	Senior Nurse	Bachelor's	N5	23	Gastrointestinal Surgery
HuN5	29	Senior Nurse	Bachelor's	N2	7	General Surgery
HuN6	37	Senior Nurse	Bachelor's	N3	14	Respiratory Medicine
HuN7	48	Senior Nurse	Associate	N3	28	Orthopedic Trauma
HuN8	39	Senior Nurse	Bachelor's	N3	17	Gastroenterology
HuN9	37	Senior Nurse	Bachelor's	N3	14	Pulmonology
HuN10	38	Senior Nurse	Bachelor's	N3	14	Orthopedics
HuN11	33	Nurse	Bachelor's	N2	10	Obstetrics & Gynecology
HuN12	35	Senior Nurse	Bachelor's	N3	13	Cardiology
HuN13	32	Nurse	Bachelor's	N2	10	Neurology
HuN14	34	Senior Nurse	Bachelor's	N3	12	Neurosurgery

The Chinese nursing competency level stratification standard divides nurses into five levels (N0–N4). In this study, N5 indicates an extended local designation for nurses with over 20 years of experience and advanced clinical responsibilities, used in some Shanghai tertiary hospitals.

### 2.3 Ethical approval and informed consent

This study was approved by the Ethics Committee of Shanghai Tenth People's Hospital (Approval No.: 22KN211). All participants provided written informed consent, participated voluntarily, and were free to withdraw at any stage. Transcripts were anonymized by replacing names with numeric identifiers to ensure confidentiality.

### 2.4 Data collection

From June 1 to July 1, 2025, the researchers conducted semi-structured, face-to-face in-depth interviews with the participants. A semi-structured interview guide was developed for data collection (see Table 2). Prior to each interview, participants were informed about the study objectives, content, methods, and confidentiality principles, and interviews were scheduled at times and locations convenient to them.

**Table 2:** Semi-structured interview guide used for data collection.

No.	Interview question
1	As a nurse working in a ward implementing no-attendant care services, what changes have occurred in your work tasks?
2	How have these changes in work content affected your job and psychological well-being?
3	Based on your observations, in what aspects have patients benefited significantly from no-attendant care services?
4	What major challenges did you encounter while promoting no-attendant care services in your ward?
5	What suggestions do you have to address the challenges and difficulties you mentioned?
6	Is there anything else you would like to share regarding no-attendant care services?

All interviews were conducted in private rooms within the hospital, with doors closed to minimize external interruptions. Only the participant and one researcher were present; no nurse managers or colleagues attended, in order to reduce potential power imbalances. A “Do Not Disturb—Interview in Progress” sign was placed outside the room, mobile phones were silenced, and a portable white noise device was used when necessary to reduce the risk of overhearing. If participants had to temporarily leave for clinical duties, the recording was paused and resumed after their return, with renewed consent. To further protect privacy and data security, interviews were not conducted in public areas such as corridors or duty rooms; all audio files and transcripts were de-identified, stored on encrypted devices, and accessible only to authorized members of the research team.

Interviews followed the framework of the guide questions, and researchers asked additional probing questions when necessary. Interviewers were not employed by the study hospitals, minimizing bias or coercion. In addition, two pilot interviews with nurses from no-attendant care wards were conducted to identify potential issues and refine the interview guide; data from pilot interviews were not included in the final analysis. Formal interviews lasted 40–60 min each. All interviews were audio-recorded with participants' consent, and transcripts were produced immediately after each session for coding and analysis.

### 2.5 Data analysis

Within 24 h after each interview, the researchers transcribed the recordings verbatim, repeatedly listened to the audio, and refined the transcripts. Filler words were retained, and notes were added to capture participants' facial expressions and demeanor when answering sensitive questions, to support subsequent interpretation and analysis.

During the design phase, we compared three common qualitative frameworks: Giorgi's descriptive phenomenology, Braun and Clarke's thematic analysis, and constructivist grounded theory. Given that this study focused on the lived experience of nurses in the no-attendant care context and emphasized traceability through participant validation, we ultimately adopted Colaizzi's seven-step method. At the same time, we incorporated systematic organization and “constant comparison” techniques from thematic analysis to enhance interpretive depth and clinical applicability.

**Figure 1:**
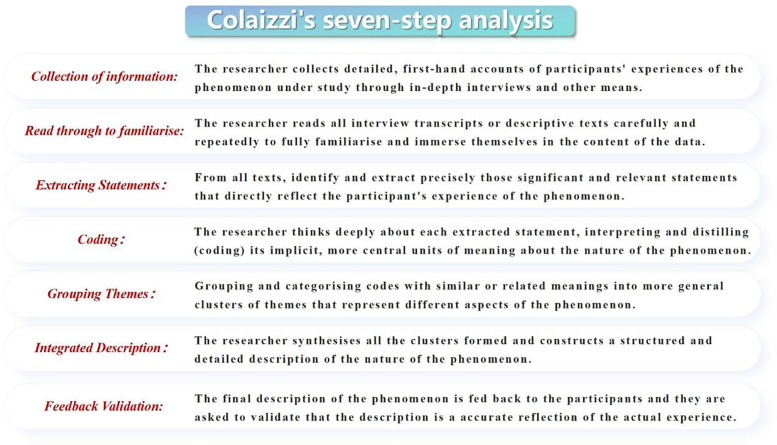
Colaizzi's seven-step analysis process used for data analysis in this study.

Using Colaizzi's seven-step method (9) (see Figure 1), themes and subthemes were extracted from the transcripts. To ensure trustworthiness, the final themes and descriptions were returned to participants for validation. Where discrepancies were identified, the researchers re-analyzed the data from step one until consensus was reached.

To enhance analytic transparency, we strictly followed Colaizzi's seven-step method and ensured traceability throughout the process. First, two researchers independently coded the transcripts line by line, extracting significant statements directly related to the research questions and segmenting them into meaning units. Second, meanings were formulated while retaining the original context, and both explicit and implicit meanings were documented. Third, similar codes were merged and grouped into subthemes, with contextual modifiers retained when necessary. Fourth, these subthemes were synthesized into interpretive subthemes and then clustered into themes, with negative cases and boundary revisions used to maintain conceptual stability. Fifth, after the initial round of coding, discrepancies were discussed by the two coders, and unresolved issues were arbitrated by a methodological expert. The refined results were then returned to participants for member checking, and modifications were made where necessary. Sixth, thematic saturation was confirmed between the 12th and 14th interviews. Throughout the process, we maintained a coding matrix and decision log, ensuring that each theme could be traced back to specific quotations.

To ensure that the themes reflected interpretive depth rather than descriptive summaries, we incorporated four mechanisms during coding and theme development: (1) reflexive memoing, which recorded researchers' assumptions, doubts, and interpretive shifts, later cross-checked against raw data; (2) constant comparison, whereby coded data were compared across and within participants to test the boundaries of concepts; (3) negative-case analysis, where divergent data were retained and used to refine theme boundaries, with “contextual modifiers” added where appropriate; and (4) peer debriefing and member checking, in which discrepancies were discussed by the research team and theme labels and descriptions were validated by participants, ensuring consistency between interpretation and data.

The following examples, all drawn from quotations already presented in the Results section, illustrate how raw statements were transformed into meaning units, codes, subthemes, and themes. For example, HuN7 stated, “We repositioned patients more than twenty times a day, and my back was so sore that I could not straighten up.” This was extracted as the meaning unit “high-frequency, physically intensive tasks,” formulated as “significant physical strain in nursing work,” coded as “high-frequency physical tasks” and “cumulative fatigue,” aggregated into the subtheme Significant Increase in Work Intensity, and ultimately subsumed under the theme Expanded Responsibilities and Intensified Workload.

As another example, HuN2 and HuN5 described, “Every shift feels like a tightly stretched string” and “It's like being made a temporary guardian,” which were extracted as the meaning units “sustained hypervigilance” and “heightened sense of moral responsibility.” These were formulated as “emotional labor coupled with a sense of unlimited responsibility,” coded as “hypervigilance,” “unlimited responsibility,” and “concentrated risk-bearing,” aggregated into the subtheme Multidimensional Accumulation of Occupational Stress, and included under the theme Expanded Responsibilities and Intensified Workload.

### 2.6 Rigor of the study

To ensure rigor, we addressed credibility, dependability, transferability, and confirmability. Credibility and confirmability were supported by building rapport with participants and triangulating qualitative data throughout transcription, analysis, and theme development. Dependability was ensured through a standardized interview protocol and detailed audit trails of the research process. Transferability was enhanced by providing rich descriptions of ward contexts, participant characteristics, and the challenges encountered during implementation of no-attendant care services.

### 2.7 Reflexivity

Some members of the research team were affiliated with institutions involved in pilot programs of no-attendant care, which may have influenced study design and interpretation. To minimize potential bias, the following measures were taken: (1) all interviews were conducted by researchers not employed in the participating hospitals, reducing concerns about hierarchical or collegial relationships; (2) team members maintained reflexive memos to document assumptions and positionality; (3) peer debriefing and review by methodological experts were used to cross-check coding and theme generation; and (4) member checking was performed by returning interpretations to participants for validation. These steps collectively enhanced the credibility and trustworthiness of the findings.

## 3 Results

**Figure 2:**
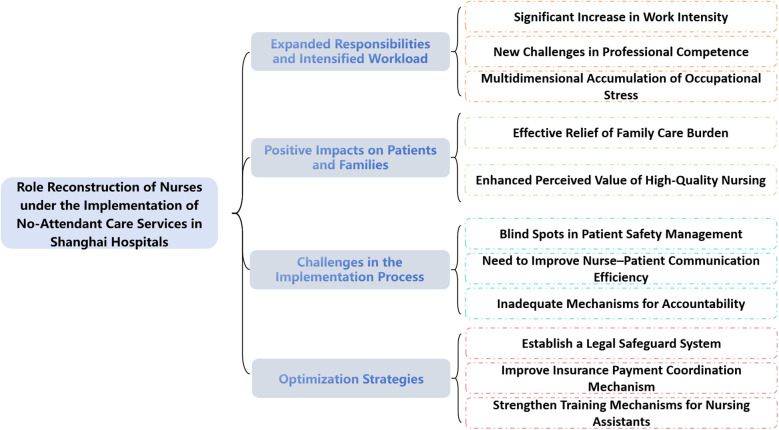
Themes and subthemes derived from Colaizzi's analysis on nurses’ role reconfiguration in a chaperone-free care service model.

This study applied Colaizzi's seven-step method to analyze the data, aiming to describe the multidimensional changes, implementation challenges, and coping strategies associated with nurses' role reconstruction under the no-attendant care model. A total of four main themes and eleven subthemes were identified (see Figure 2).

### 3.1 Expanded responsibilities and intensified workload

#### 3.1.1 Significant increase in work intensity (physical strain in the absence of family caregivers)

Participants generally reported that after the implementation of no-attendant care, the absence of family caregivers required nurses to “fill the gap” with their own physical labor. Basic care and bedside monitoring became more concentrated, resulting in sustained physical exhaustion and insufficient recovery. Examples are provided below.

HuN1 stated: “Most patients in the neurology ward are unable to care for themselves. Now I not only have to perform basic care but also stay vigilant to prevent accidents. From morning till night it's nonstop, and sometimes I don't even have time for lunch.”

HuN7 stated: “Turning orthopedic patients is not only a skill but also heavy physical work. Without family members, it's up to us and a few assistants. In a single day, I may reposition patients more than twenty times, and my back is so sore I can't straighten up.”

Summary: This experience was not only about increased tasks but also reflected the tension nurses faced between physical limits and professional responsibility when substituting for family caregivers.

#### 3.1.2 New challenges in professional competence (reconfiguration of knowledge and communication skills)

With families absent, patients' needs for rehabilitation guidance and illness explanation were directed more fully to nurses. This pushed nursing beyond “task execution” toward a dual emphasis on knowledge updating and health education, highlighting the reconfiguration of relational skills such as clinical observation and communication. Examples are provided below.

HuN4 stated: “In the past, it was enough just to follow procedures. But now, without family members, patients and families want much more guidance. One patient asked how to plan a diet scientifically after surgery, and I realized old experience was not enough, so I had to keep learning new nutrition knowledge.”

HuN13 stated: “Neurology patients change rapidly, and without family present we must detect problems more sensitively. And all the communication with patients and families falls to us. We have to learn to explain complex illnesses in simple language, and our communication skills are being tested like never before.”

Summary: Nurses shifted from being mere task executors to integrators of information and meaning, reflecting the coordinated evolution of professional knowledge and communication capacity.

#### 3.1.3 multidimensional accumulation of occupational stress (hypervigilance, concentrated risk, and moral responsibility)

Participants described stress accumulation across three interwoven dimensions: (1) workload–pace stress from combined basic care, frequent rounds, and emergency responses; (2) risk and decision-making pressures concentrated on nurses due to the absence of “secondary supervision” by families; and (3) emotional labor driven by sustained hypervigilance and a sense of “unlimited responsibility.” Examples are provided below.

HuN5 stated: “The stress is overwhelming. On the one hand, the work itself is dense, and I fear missing something. On the other hand, it's this sense of ‘unlimited responsibility.’ With no family around, it's like we are given the hat of a ‘temporary guardian.’”

HuN2 stated: “In the respiratory ward, many patients are critically ill. With no family members, all emergencies fall to us. One night a patient suddenly had difficulty breathing, and I was so nervous that even after handling it, I couldn't calm down. Every shift feels like a tightly stretched string.”

Summary: Stress was not only a result of task intensity but also revealed the existential tension of moral responsibility and hypervigilance in the absence of family witnesses. (In a few wards with adequate assistant staffing or more independent patients, nurses reported relatively less psychological tension and physical strain.)

### 3.2 Positive impacts on patients and families

#### 3.2.1 Effective relief of family care burden (institutional transfer of care responsibility)

No-attendant care, through professionalized institutional supply, reduced families' long-term time and financial burdens, shifting responsibility from “continuous family presence” to “organized institutional provision.” This allowed patients and families to rebalance care with work and daily life. Examples are provided below.

HuN14 stated: “I met a patient from out of town whose family was staying in a cramped hotel nearby, which was very costly. With no-attendant care, they no longer needed to stay all the time. One family member told me the money saved covered two months of their child's living expenses. That really helps ordinary families.”

HuN11 stated: “For gynecology patients, having family present is sometimes inconvenient. Now with no-attendant care, patients can rest assured, and families don't feel awkward. One patient's husband said he could finally go on business trips without worrying, balancing work and family, and he was very grateful.”

Summary: Relief of family burden was reflected not only in finances and time but also in enabling patients to focus more on rehabilitation and reshaping interaction channels with the nursing team.

#### 3.2.2 Enhanced perceived value of high-quality nursing (salience of early risk identification and systematic basic care)

In daily practice, participants noted that the “perceived value” of early risk identification and systematic basic care became more visible to patients and families, highlighting the distinctiveness of professional nursing. Examples are provided below.

HuN4 stated: “Stoma complications decreased from 15% to 5% because we assessed mucosal color and secretions promptly. Families could change ten bags without noticing problems, but yesterday I spotted early ischemia and handled it, preventing necrosis.”

HuN9 stated: “For long-term bedridden patients in respiratory wards, we turn them regularly and perform chest percussion to prevent pressure sores and pneumonia. A family member told me they used to worry they weren't doing it right, but now with professional nurses and assistants, they feel reassured.”

Summary: This “visible professionalism” generated value feedback and reaffirmed nurses' professional identity, supporting the positive shaping of the nursing profession.

### 3.3 Challenges in the implementation process

#### 3.3.1 Blind spots in patient safety management (risk exposure without family witnesses)

The absence of families as “co-witnesses” weakened immediate reminders and self-restraint, leading to frequent non-adherent behaviors such as unsupervised ambulation or self-medication. Safety management shifted from “multi-point supervision” to “single-point pressure,” amplifying risk exposure.

HuN7 stated: “An elderly patient hid his antihypertensive medication and fainted in the bathroom from low blood pressure at night. Luckily, I found him on rounds. Who would bear responsibility if he was injured? Now we have to watch them swallow pills, but patients with high blood sugar still sneak sweets, which makes it impossible to guard against everything.”

HuN8 stated: “Some patients think they are strong enough and ignore advice, insisting on moving around alone. When family members were present, they could intervene. Without them, I've had several near falls. One patient slipped going to the toilet when we weren't watching, and fortunately there was no serious harm.”

Summary: The essence of these safety challenges lies in the reconfiguration of supervisory structures, requiring new multi-point, closed-loop management through processes and technology.

#### 3.3.2 Need to improve nurse–patient communication efficiency (relational labor in extended communication chains)

With no-attendant care, patients' and families' needs for information and emotional support were directed more fully to nurses, while reliance on phone or video communication lengthened the communication chain. Nurses reported repeated explanations of medical information, coupled with relational and emotional labor, resulting in dual burdens of workload and emotional strain.

HuN5 stated: “Some families could only get updates by phone or video. Communication was exhausting.I once spent a long time explaining a patient's recovery, but the family still questioned our plan, and I nearly lost my voice.”

HuN10 stated: “For patients with limited education, it was extremely difficult. We used metaphors and examples until they understood, but then their family called again, and we had to explain everything once more.”

Summary: The challenge was not merely transmission of information but the amplification of relational labor, resulting in dual strains on efficiency and emotion. (In a few cases, when families had higher health literacy and a designated point of contact, remote communication was smoother and burdens were lighter.)

#### 3.3.3 Inadequate mechanisms for accountability (Longer collaboration chains and concentrated responsibility)

With both nursing assistants involved and family members absent, collaboration chains lengthened while accountability became concentrated on nurses. When incidents occurred, disputes over role boundaries became more frequent.

HuN3 stated: “When something happened to a patient in the ward, families insisted it was the nurse's fault. Once a patient injured himself accidentally, but the family said it was because we didn't care properly. With so many patients, we can't watch every one of them, but responsibility was still unclear.”

HuN12 stated: “A patient aspirated while being fed by an assistant, and the family accused us of poor supervision. I had ten patients to manage. How could I monitor every bite? It was really unfair.”

Summary: The crux of accountability disputes lay not in generic “inadequate nursing,” but in the redistribution of supervisory and accountability structures caused by family absence.

### 3.4 Optimization strategies

#### 3.4.1 Establish a legal safeguard system (clear responsibility boundaries and predictability)

Participants emphasized the need for specific regulations clarifying service content and role boundaries, thereby establishing predictable accountability rules and procedural justice to sustain implementation.

HuN6 stated: “Nursing work is high risk, and we cannot continue without legal protection. We hope for specific laws on no-attendant care services, clearly defining content and responsibilities, so we have a basis for our work and families can understand.”

HuN7 stated: “For unexpected events, laws should define accountability. For example, when conditions suddenly worsen due to disease vs. when outcomes stem from inadequate nursing, there must be standards so we will not feel constrained.”

Summary: Legal safeguards are not supplementary but prerequisite conditions for predictable accountability and nurses’ professional security.

#### 3.4.2 Improve insurance payment coordination mechanism (equity and incentive compatibility)

Integrating no-attendant care services into medical insurance or long-term care insurance could improve accessibility and incentive compatibility: reducing patients' financial burden, narrowing inequalities, and promoting standardization and quality improvement through policy levers.

HuN8 stated: “Insurance reimbursement would increase acceptance. One patient told me that if part of the cost could be covered, he would definitely choose professional care rather than troubling his family, and he hoped the policy would be implemented soon.”

HuN11 stated: “Including no-attendant care in insurance would promote fairness. Regardless of financial status, every patient could access professional nursing services, which aligns with the original intent of health insurance.”

Summary: Payment coordination is a critical link for scaling the model, directly tied to equity, sustainability, and quality governance.

#### 3.4.3 Strengthen training mechanisms for nursing assistants (task allocation and competency loops)

Participants highlighted the need for systematic training for nursing assistants in emergency skills, specialized care, and communication, enabling clear task allocation and competency loops that support nurses' professional focus while enhancing overall team capacity.

HuN9 stated: “Improving assistants’ competence benefits the whole team. If they are more professional, we can concentrate on patient monitoring and advanced care, and patients receive better service.”

HuN13 stated: “Some assistants lack emergency knowledge. Once a patient choked during feeding, and the assistant froze, only calling for help. If they had systematic emergency training, they could have responded immediately.”

Summary: Building dual loops of competency and task allocation fosters a collaborative structure where nurses focus on advanced care and assistants competently deliver basic care.

## 4 Discussion

### 4.1 Role reconstruction and revalorization of nursing under No-attendant care

The findings of this study indicate that the implementation of the no-attendant care model has played a positive role in optimizing nursing services and enhancing patient experience, while also triggering profound reconstruction of nursing roles. Nurses not only continued to assume traditional clinical and technical care tasks, but also took full responsibility for basic daily care, rehabilitation guidance, psychological support, and health education. This multidimensional expansion of roles reflects the dynamic extension of nurses' skill boundaries and interactive capacities in demanding contexts. Viewed through the lens of Benner's Novice to Expert framework, this can be interpreted not as a linear progression through predefined stages, but as an experiential enhancement of clinical judgment and holistic care ability, cultivated in complex and uncertain situations (10). Based on our data, such expansion primarily manifested in three experiential structures: bodily workload, concentrated responsibility and risk, and amplified relational–emotional labor.

During interviews, many nurses reported that under no-attendant care, they had to detect changes in patients' conditions more promptly and accurately, respond patiently to the concerns of patients and families, and employ individualized, systematic care strategies to reduce complications. For example, participants cited the early detection of stoma complications and systematic repositioning and chest percussion in respiratory wards as evidence that the “perceived quality of basic nursing care” became more salient in the no-attendant care context. Such accounts resonate with Radwin et al.'s findings that patient-centered nursing, through proactive assessment and timely intervention, significantly enhances patient satisfaction and adherence (11), here serving as an interpretive reference consistent with our data.

The no-attendant care model not only alleviated families' financial and psychological burdens, allowing patients to focus more on rehabilitation, but also reshaped nurse–patient interactions. With families no longer continuously present, patients directed their informational, emotional, and care-related needs more directly to nurses, resulting in more frequent and deeper one-to-one exchanges. Over time, this fostered trust and dependence, strengthening the therapeutic relationship. This mechanism echoes Watson's Theory of Human Caring, which emphasizes relational care (12); in this study, we regard it as a contextualized manifestation of caring practice under no-attendant care, rather than a direct verification of a universal caring essence.

At the same time, the continual expansion of roles posed new challenges. Nurses faced increasingly complex clinical issues, higher care demands, and diverse societal expectations, leading to marked increases in occupational stress. In this study, nurses consistently expressed a sense of “unlimited responsibility,” a perception consistent with findings from domestic and international studies. For example, Beljikangarlou et al. reported that nurses in no-attendant wards often experience heavy psychological burdens from high-intensity workloads and frequent emergencies, with some showing signs of burnout or physical symptoms (13). Similarly, Kim et al. in Korea found that nurses in no-attendant settings had to independently assume greater medical and emotional support responsibilities during acute events, which promoted professional growth but significantly increased physical and mental strain (14).

From the perspective of role evolution, this shift reflects a transformation from “task-oriented” to “holistic health-oriented” nursing thinking. Nurses are no longer simply executors of basic tasks, but integrators of technical, emotional, educational, and coordinative functions, serving as full-spectrum health managers throughout hospitalization. Such transformation requires continuous knowledge updating, enhanced clinical judgment, and comprehensive competencies to adapt to rapidly changing contexts.Without adequate organizational support, such as sufficient staffing, psychological support mechanisms, and interdisciplinary collaboration platforms, nurses may remain in a state of prolonged high stress and heavy workload, which threatens sustainable professional development and the quality of nursing care (15). It should be noted that the references to Benner and Watson in this study are used as interpretive lenses driven by data, rather than as theoretical validation. Future work could further explore the phenomenological essence of caring and professional growth.

Therefore, institutional measures should be taken to strengthen nurses' psychological support and protection systems, establish routine mechanisms for emotional relief and stress reduction, and optimize staffing and scheduling to safeguard nurses' well-being and professional sustainability. Coupled with multi-level and multi-channel continuing education and support networks, these measures would enhance nurses' resilience and competence under the no-attendant care model, and promote the evolution of nursing services toward higher quality and stronger humanistic caring.

### 4.2 Challenges in the implementation process of No-attendant care

This study further revealed prominent issues in the practical operation of the no-attendant care model, particularly regarding patient safety, communication efficiency, and accountability.

First, patient safety risks increased significantly. Without the supervision and assistance of family caregivers, patients were more prone to engage in non-adherent behaviors such as unsupervised ambulation or self-medication, which in turn led to falls, hypoglycemia, hypotension, and other adverse events. Several participants noted that patients often lacked self-management ability and vigilance, requiring nurses to conduct more frequent rounds and real-time monitoring. These findings are consistent with Wang et al., who reported that in the absence of family caregivers, the incidence of falls and other adverse events rose markedly among hospitalized elderly patients, particularly those with cognitive impairment or multiple comorbidities (16). From an experiential perspective, this heightened risk was not merely an accumulation of isolated incidents, but rather a contextual manifestation of “single-point burden bearing” following the reconfiguration of supervisory structures.

To address these risks, participants suggested combining advanced technological approaches, such as bedside smart monitoring systems and wearable devices for vital signs, to detect abnormal behaviors or physiological changes promptly. At the same time, optimizing the frequency of ward rounds and strengthening collaboration between nurses and nursing assistants could help form a “multi-point, closed-loop” safety management model to prevent incidents. This direction aligns with the logic of “multi-point, closed-loop” safety management presented in our Results, aiming to rebuild a framework of “multi-stakeholder co-governance” through processes and technology.

Second, communication barriers were particularly prominent. Without family presence in the wards, families relied on telephone or video calls to obtain updates, often resulting in distorted information transfer, inefficiency, and reduced trust. These challenges were especially evident among patients and families with limited educational backgrounds, where medical terminology was difficult to understand. This led to repeated explanations, misunderstandings, and even skepticism about nursing plans, thereby adding to nurses' communication burden and emotional stress. These findings are consistent with Rouleau et al., who highlighted the dilemma of remote nursing communication in the digital era and noted that achieving efficient, accurate, and compassionate communication remains a pressing challenge (17). This corresponds to the theme in our Results of “relational labor in extended communication chains,” underscoring that communication burdens themselves are a visible component of care that require resource allocation. To mitigate this, it is necessary to strengthen nurses’ skills in health education and communication, develop standardized and visualized patient education materials, reduce technical jargon, and improve patient and family understanding of nursing interventions and treatment plans. Additionally, scenario-based simulation and communication training could enhance nurses' ability to cope with complex communication scenarios and foster stronger nurse–patient trust.

Finally, unclear accountability mechanisms represent another major institutional challenge of the no-attendant care model. Many nurses reported that when unexpected events occurred or when incidents arose during collaboration with nursing assistants, families often attributed all responsibility to nurses, resulting in heavy professional and psychological pressure. This reflects the lack of explicit legal frameworks and standardized accountability mechanisms tailored to the no-attendant model, limiting nurses' professional autonomy and undermining their sense of security and motivation. Blegen et al. have emphasized that clear legal boundaries and standardized accountability mechanisms are essential to safeguard nurses' professional security and reduce burnout (18). In other words, the crux of these disputes lies in the redistribution of supervisory and accountability structures, namely concentrated accountability and longer collaboration chains, rather than simply in “inadequate nursing.”

By comparison, in systems such as the United Kingdom's National Health Service (NHS), clear regulations stipulate that nurses should not be held fully responsible for risks arising from patients' non-adherent behaviors (e.g., unsupervised ambulation, self-medication), provided that nurses have fulfilled their obligations of adequate monitoring and sufficient information disclosure. Such legal and institutional support not only protects nurses’ rights but also reduces disputes and unnecessary blame, thereby promoting the stability and sustainability of nursing services.

Taken together, these findings highlight that synchronizing institutional frameworks and financial mechanisms is key to shifting the burden from “individual nurses” to “system-level support.”

In conclusion, although the no-attendant care model plays an important role in alleviating family caregiving burdens and enhancing the professionalization of nursing services, its full implementation requires improvements in institutional frameworks, legal safeguards, intelligent management, and humanistic communication. Insurance support also remains a critical factor for broader adoption. At present, most regions have not included related costs under reimbursement, limiting patients' and families' acceptance and affecting service equity. Future efforts should explore integrating these services into medical or long-term care insurance, with clearly defined service contents and standardized payment mechanisms, to improve accessibility and provide institutional security for the nursing workforce.

## 5 Limitations

In this study, the sample consisted mainly of senior nurses, while early-career nurses, including probationary and student nurses, were not included. As a result, the study may have missed unique experiences related to uncertainty, lack of confidence, and coping strategies at the early stage of professional development. Therefore, the findings primarily reflect the perspectives of experienced nurses. Future studies should incorporate nurses at different career stages to compare how role reconstruction varies by seniority and professional development.

In addition, most participants were at level N3 or above. The exclusion of probationary nurses may have omitted perspectives on stress perception, self-evaluations of competence, and collaboration strategies during the early stages of practice. Future research should include a broader range of professional levels to examine the heterogeneity of role reconstruction across different stages of nursing careers.

## Data Availability

The original contributions presented in the study are included in the article/Supplementary Material, further inquiries can be directed to the corresponding author.
